# Dominance-related seasonal song production is unrelated to circulating testosterone in a subtropical songbird

**DOI:** 10.1016/j.ygcen.2016.05.011

**Published:** 2016-07-01

**Authors:** Jenny E. York, Andrew N. Radford, Bonnie de Vries, Ton G. Groothuis, Andrew J. Young

**Affiliations:** a Centre for Life and Environmental Sciences, University of Exeter, Penryn Campus, Cornwall TR10 9EZ, UK; b School of Biological Sciences, Life Sciences Building, 24 Tyndall Avenue, Bristol BS8 1TQ, UK; c Behavioural Biology, The Groningen Institute for Evolutionary Life Sciences, University of Groningen, The Netherlands

**Keywords:** Tropical endocrinology, Sexual signalling, Circulating testosterone, Seasonality, Dominance

## Abstract

Circulating testosterone (T) is widely considered to play a key role in the production of sexual displays by male vertebrates. While numerous studies support a role for circulating T in promoting the production of song in male birds, this understanding is based primarily on evidence from seasonally breeding northern temperate species, leaving it unclear whether this mechanism generalizes to other regions of the world. Here we investigate whether variation in circulating levels of T can explain the marked within- and among-individual variation in male song performance observed in a subtropical population of the year-round territorial white-browed sparrow weaver (*Plocepasser mahali mahali*). Our findings reveal that both circulating T and male song production peaked at a similar time point, halfway through the population-level breeding season. However, while dominant males were more likely to sing and sang for longer than subordinate males, within-group paired comparisons revealed no dominance-related differences in circulating T. Moreover, comparisons both among and within individual dominant males revealed that song duration, syllable rate and proportion of time spent singing were all unrelated to circulating T. Together, our findings suggest that natural variation in male song production, at least in this population of white-browed sparrow weavers, is achieved principally through mechanisms other than variation in circulating T concentration. More widely, our results are in line with the view that male song production is not exclusively regulated by gonadally synthesized steroids.

## Highlights

• Whether testosterone (T) regulates song in subtropical birds is poorly known.
• Subtropical white-browed sparrow weavers show seasonal profiles in T and song.
• Dominant males sang more than subordinate males, despite comparable T.
• Dominant male song production was not correlated with circulating T.
• We highlight the need to consider the role of alternative neuroendocrine mechanisms.

## 1 Introduction

Male vertebrates produce a wide variety of sexually selected behaviours to repel rivals or attract mates and a central question in evolutionary endocrinology is whether the mechanisms that regulate the expression of such behaviours are conserved or divergent across species (Hau, 2007, Hau et al., 2008, Hau et al., 2010). In particular, the circulating androgen testosterone (T) has been a key focus of research seeking to understand how male sexual signalling behaviours are regulated (Ball, 1999, Fusani, 2008, Garamszegi et al., 2008). While there is a weight of evidence supporting a fundamental role for circulating T in male sexual signalling behaviour, there has been a significant bias towards northern temperate zones in the species studied (Garamszegi et al., 2008, Goymann et al., 2004, Hau et al., 2008, Wingfield et al., 2013).

Fundamental differences in life-history traits between temperate and tropical birds have long been recognised (Stutchbury and Morton, 2008, Macedo and Machado, 2013). Recent research also suggests that the underlying physiological mechanisms associated with life-history trade-offs exhibit similar latitudinal contrasts (Martin et al., 2004, Garamszegi et al., 2005, Owen-Ashley et al., 2008, Wingfield et al., 2007). Together, this evidence has led to the generalisation that birds in the tropics exhibit a slower ‘pace of life’ than their temperate counterparts (Wikelski et al., 2003) and indicates the importance of considering latitudinal contrasts when investigating the evolution of endocrine mechanisms in the context of life-history theory (Hau, 2007, Hau et al., 2010). Despite their potential value, however, detailed empirical investigations of the role of circulating T in regulating investment in sexually selected behaviours remain rare for tropical species and rarer still from those inhabiting the subtropics (Hau et al., 2008).

Early studies of circulating T in tropical passerines revealed average circulating T concentrations an order of magnitude lower than those of temperate birds (Levin and Wingfield, 1992, Hau, 2007, Hau et al., 2008, Hau et al., 2010). Furthermore, circulating T levels remain low throughout the breeding season in many tropical species, whereas temperate species typically exhibit testosterone peaks during a brief breeding window (Levin and Wingfield, 1992, Hunt et al., 1995, Hau et al., 2000, Wikelski et al., 2003); a limited number of within-species comparisons support this latitudinal pattern (Rödl et al., 2004, Goymann et al., 2006, Wingfield et al., 2007; but see: Moore et al., 2002, Ryder et al., 2011). While numerous potential ecological drivers of latitudinal patterns have been proposed (Cardillo, 2002, Garamszegi et al., 2008), key among these with regard to circulating T may be the extent of seasonality in territorial behaviour and its implications for seasonal patterns of social instability (Hunt et al., 1995). Many temperate species are exposed to brief windows of intense intra-sexual conflict over access to mates and territory establishment, and circulating T is thought to play a key role in upregulating these behaviours (such as male song production and aggression) during these periods (Wingfield, 2012). By contrast, tropical and subtropical zones may include a higher proportion of year-round territorial species and this lifestyle may be associated with different seasonal patterns in circulating T (but see: Ryder et al., 2011).

Male bird song is one of the most extensively studied vertebrate sexual signalling systems, both in the laboratory and in ecologically relevant contexts, and therefore provides an excellent opportunity for latitudinal contrasts (Ball, 1999, Catchpole and Slater, 2008, Gahr, 2014). Correlative evidence from wild temperate birds indicates that population-level elevations in circulating T in spring are coincident with population-level elevated song expression (Foerster et al., 2002, Van Duyse et al., 2003). Furthermore, experimental manipulations have revealed that artificially induced variation in circulating T levels can indeed influence the production of song by males in both laboratory and wild birds (Table 1). Similarly, studies that experimentally inhibited T reception have largely shown the opposite effect of reducing song production (Table 1). However, demonstrating that song production is altered by manipulating circulating T and/or T reception does not, in isolation, confirm that natural variation in song production is actually achieved in the wild through variation in the levels of circulating T, given that plausible alternative mechanisms exist (Soma et al., 2008). It is therefore important to also investigate whether natural within- and among-male variation in song production is predicted by natural variation in circulating T in wild populations. While rather fewer studies have investigated this association during breeding periods, there is some evidence of positive correlations between circulating T and song from wild populations of northern temperate birds (Table 1). There is, however, a conspicuous lack of similar studies investigating the link between circulating T and song production in wild populations of tropical and subtropical songbirds (Table 1).

Here, we investigate whether natural variation in circulating T predicts natural variation in male solo song production in a subtropical population of wild white-browed sparrow weavers (*Plocepasser mahali)*; a year-round territorial songbird that is distributed across sub-Saharan Africa. White-browed sparrow weavers live in cooperatively breeding groups of 2–12 individuals, in which a dominant female entirely monopolises maternity, a dominant male largely monopolises paternity (12–18% of paternity is lost exclusively to extra-group dominant males), and subordinates of both sexes help to rear their young (Harrison et al., 2013a, Harrison et al., 2013b). Males produce solo song at dawn during the breeding season almost exclusively at dawn (Voigt et al., 2007, York, 2012, York et al., 2014). Previous studies of this species suggest that the mean circulating T concentrations of males may vary in relation to latitude, as while very low mean circulating T concentrations were first reported in a tropical subspecies (*P. m. pectoralis*, Northern Zambia; Wingfield et al., 1991), higher concentrations were found in a population of the more southerly subspecies (*P. m. mahali,* Southern Zimbabwe; Voigt and Leitner, 2013). Studies of the latter sub-species suggest that circulating T does play a role in the development and initial expression of male solo song production, based on the stimulatory effect of experimentally induced T elevation in females (Voigt and Leitner, 2013), and have revealed pronounced dominance-related differences in male solo song production (subordinate males were not known to sing in the Zimbabwean population; Voigt et al., 2007). Whether these dominance-related differences in solo song production are associated with dominance-related differences in circulating T remains unclear, as dominance-related differences in T have been detected in the more northerly subspecies for which solo song studies are lacking (Wingfield et al., 1991) but were less clear in studies of the more southerly subspecies in which these song patterns were recorded (Voigt and Leitner, 2013, Voigt et al., 2007). The matched sampling of T levels and solo song production is required to establish whether the marked among and within-individual variation in male song production in this species is indeed associated with natural variation in their circulating levels of T.

Specifically, we test three sets of predictions that would be made if male solo song production was regulated by variation in circulating T in a concentration-dependent manner. We do so using our subtropical South African study population of *P. m. mahali*, which lies at a more southerly latitude than both the Zambian and Zimbabwean populations studied to date (see above). First, we investigate whether the circulating T levels and solo song production of dominant males show comparable patterns of temporal variation over the course of the breeding season (as has been reported in temperate birds; Foerster et al., 2002). Second, we investigate whether dominant and subordinate males in this most-southerly population also differ in their solo song production characteristics and whether such differences are mirrored by dominance-related differences in circulating T. Third, we investigate whether natural variation in the circulating T levels of dominant males predicts natural variation in their solo song performance characteristics (performance duration, syllable rate and proportion time spent singing) using both among- and within-male contrasts.

## 2 Methods

### 2.1 General methods

**Fig. 1:**
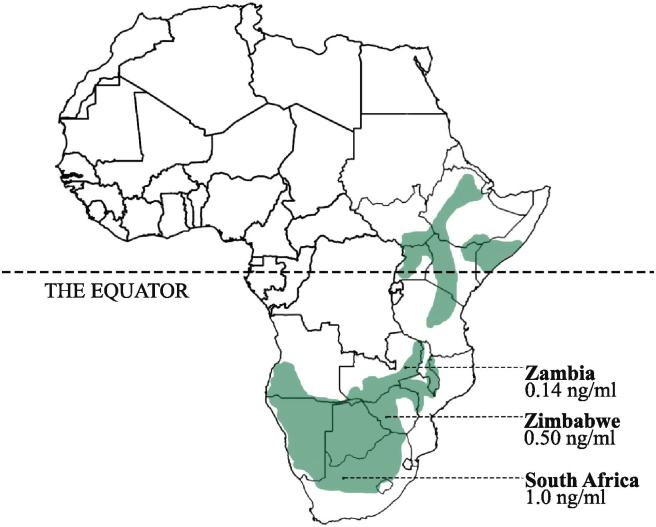
The geographic range of white-browed sparrow weavers, *Plocepasser mahali*, (shaded areas) and the mean circulating testosterone concentration of males in three distinct populations measured during the breeding season (October-March): Zambia (mean calculated from figures in Wingfield et al., 1991; n = 81 samples), Zimbabwe (mean calculated from figures in Voigt and Leitner, 2013; n = 47 samples), and South Africa (mean from data in this study; n = 133 samples).

This field study was conducted between October 2010 and April 2011 in the context of a long-term project monitoring a population of over 40 cooperatively breeding groups of white-browed sparrow weavers at the Tswalu Kalahari Reserve, South Africa (27°15′ S, 22°26′ E, elevation: 1195 m; see Fig. 1 for species geographic range). All individuals in the population have unique colour-ring combinations for identification purposes (Harrison et al., 2013a, Harrison et al., 2013b, York et al., 2014). Males were identified during the dawn chorus using their ring combination and a black dye mark applied to feathers on the lower abdomens of dominant males during routine captures. Dye marks were used as an additional criterion to confirm individual identification, as the low light levels at dawn can reduce the reliability of colour-ring discrimination (York, 2012, York et al., 2014). Male dominance status was assigned based on weekly assessments of behaviours following previous studies (Collias and Collias, 1978, Harrison et al., 2014, Cram et al., 2015) including the chasing and displacement of other males, and the duetting with and guarding of the dominant female (Harrison et al., 2013a, Harrison et al., 2013b, York et al., 2016, Cram et al., 2014).

### 2.2 Male solo song observations

Observation sessions began 2 h before sunrise, which is well before the earliest dawn song performance has been recorded to start in this population (York et al., 2014). The dawn solo song performance typically begins in the roost chamber and the male then emerges to continue singing around the territory from one or more song perches, until the performance ends shortly before sunrise. Performance “start time” was defined as the time at which the first notes of dawn song were produced, and performance “end time” as the time at which the last notes of dawn song were produced. Total “performance duration” was calculated as the difference between these times. Males were continuously observed from their performance start time (typically in the roost chamber) until they could be visually identified using their colour rings and feather dye mark and any occasions where male identity could not be confirmed were excluded from the analyses.

### 2.3 Song file recordings and song analysis

**Fig. 2:**
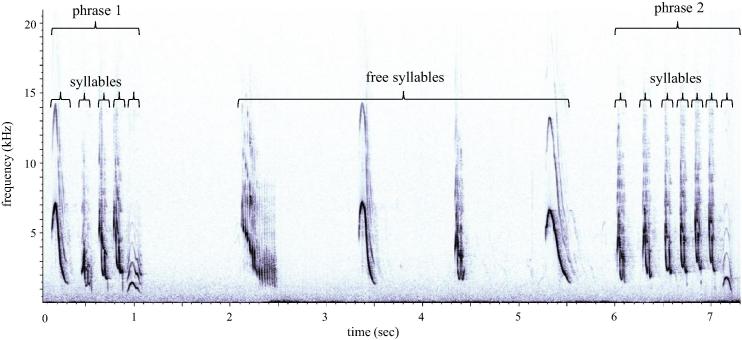
A spectrogram of dominant male white-browed sparrow weaver dawn solo song recorded in South Africa. The song has a variable structure that consists of ‘free’ syllables uttered alone and syllables arranged together in phrases (within a phrase, inter-syllable intervals are no longer than 200 ms; Voigt et al., 2007).

Recordings of pre-emergence song (song produced prior to the male’s emergence from his roost chamber) were made from within 10 m of the male, using a Sennheiser ME66 directional microphone with a K6 power module (2004 Sennheiser), and a Marantz PMD660 solid-state recorder (D&M Holdings Inc.). Avisoft-SASLab Pro 5.1.16 (R. Specht, Berlin, Germany) was used to generate spectrograms (Hamming window, FFT of 1024 points, time resolution of 5.8 ms and 50% overlap). We focused our attention on song performance characteristics that previous studies have most commonly found to be impacted by the manipulation or blockade of circulating T (see Table 1 for examples). Male song has a variable structure that consists of syllables produced either alone or arranged in phrases (see Fig. 2 for an example; Voigt et al., 2007). “Syllable rate” (number of syllables/min) and “proportion of time spent singing” (cumulative duration of syllables) were calculated for the 5-min sample period following the performance start time (during which time the male remained within his roost chamber). This sample period was selected as a standard time point during the song performance for contrasts within and between males, and because males were guaranteed to be stationary during this period, ensuring that the quality of recording files was consistent.

### 2.4 Capture and blood sampling

Captures were conducted at night, prior to the dawn chorus, whilst the males were in their roost chambers, by flushing them into a customised catching net (SAFRING license 1444). Males could not be caught at dawn as they were highly sensitive to disturbance immediately before and during song production. Therefore, to avoid effects of capture on our assessments of male song characteristics, males were captured for blood sampling on the night following their matched dawn song recording session. A blood sample was taken from the brachial vein with a 26 g needle (approx. 160 μl whole blood was collected). All samples were collected rapidly following capture (duration from initiation of capture to the end of sample collection: mean ± SD = 5.8 8 2.4 min) and the capture-to-bleed lag duration was never found to be significant in the analyses of circulating T (see Results), therefore it is unlikely that capture stress induced a rapid increase in testosterone that would influence our findings (Van Hout et al., 2010, Deviche et al., 2012). Blood sample collection took place between 20:00 and 05:00; time of sampling (the time elapsed since sunset, to standardise for effects of seasonal variation in day length) was never found to be significant in the analyses of circulating T (see Results). Furthermore, there was no significant difference in T concentrations between a set of blood samples collected from dominant males on three evenings (21:00 – midnight; n = 9 males; T concentration mean ± SD = 0.90 8 0.64 ng/ml) and a set of samples collected on the three ensuing dawns before the dawn chorus (03:00–05:00, n = 10 males, T concentration mean ± SD 8 0.58 ± 0.28 ng/ml; Welch two sample *t*-test: t = 1.35, p = 0.20). To allow paired comparisons of the T concentrations of dominant and subordinate males within the same group, blood samples were collected from both individuals on the same night. Whole blood samples were immediately separated in the field via centrifugation (12,000*g* for 3 min; Haematospin 1400; Hawksley Medical and Laboratory Equipment). The plasma was then drawn off and stored initially on ice (for 1–4 h), before being transferred to liquid nitrogen on return to the field base. The plasma samples were then shipped internationally on dry ice and stored at −80 °C until analysis five months later.

### 2.5 Testosterone analysis

T was extracted from the plasma following a previously validated protocol (Goerlich et al., 2009). Briefly, the samples were extracted twice using diethyl-petroleum ether, 70:30 (vol/vol), snap-frozen, dried under a stream of nitrogen and then stored in a −20 °C freezer until a single assay was conducted for all samples. T concentration was then determined via a single coated tube competitive-binding radioimmunoassay (Orion Diagnostica, Spectria: 68628; detection limit: 0.02 ng/ml; cross-reactivity: 100% with testosterone, 4.5% with 5α-dihydrotestosterone, 0.007% with androstenedione). The average recovery rate (81.7%) for T was calculated by counts of tritiated T (20 μl), which was added to the initial plasma sample before the extraction step. All T values were adjusted for the recovery rate for the focal sample. Pooled samples were used to create dilution curves so that parallelism could be confirmed, all samples were analysed in a single assay, and the intra-assay coefficient of variation was 3.03%.

### 2.6 Statistical analysis

All analyses were conducted using R 2.14.1 (R Development Core Team). Linear Mixed Models (Bates et al., 2009) started with a model including all of the fixed terms and interactions of interest (maximal model), followed by stepwise removal of each term in the order which resulted in the least significant change in deviance using a likelihood-ratio test for model comparison, until the minimal adequate model was found (when only significant terms remained; Crawley, 2007). The significance of explanatory variables was determined by testing for the change in deviance in the fit of the model when the term was removed from the model. Repeated measures of groups and individuals were controlled for as required, by fitting group and individual identity as random terms, as detailed below. The residuals were checked for normality and homoscedasticity, and if they did not meet these assumptions the response terms were transformed to resolve this (see details below).

For each of the five main models detailed below, each data point was attributed a ‘season day’, with season day 1 being October 1st 2010. Season day was fitted as both a quadratic (abbreviated to: season day^2^) and a linear (season day) term, to allow for the predicted curved relationship between the date during the season and the two response terms (circulating T and song production). Wherever season day^2^ proved significant, both the quadratic and the linear term were retained in the model (Crawley, 2007).

#### 2.6.1 Does song production vary in relation to dominance status and timing during season?

A generalised linear mixed model (GLMM) with binomial error was used to investigate the probability of dawn song production by dominant and subordinate males. On 216 mornings over the course of the breeding season (October to April), and at a total of 24 social groups that contained one or more adult subordinate males (>1 year old) at the time of sampling, it was determined whether the dominant male sang (binomial response: 0 = did not sing; 1 = sang) and whether one or more subordinate males in the group sang (0 = did not sing; 1 = one or more sang). As such, the dataset for this analysis contained two lines per group per observation session, which were assigned a unique ‘session ID’ number; both ‘session ID’ and ‘group ID’ were included as random terms.

A paired analysis was also conducted to establish whether dominance status predicts dawn song performance duration. The performance durations of the dominant male and his singing subordinate male were calculated during the same dawn session (so as to control for variance in song production arising from either the day or location of sampling) for 10 social groups, where song performance duration was determined for both males.

#### 2.6.2 Does circulating T vary in relation to dominance status and timing during season?

A general linear mixed effects model (LMM) was used to investigate whether a male’s dominance status predicts his circulating T concentration. T values were square-root transformed for analysis. The time lag in minutes between capture onset and the end of blood sample collection was included as a possible covariate to control for any effect of this duration on T concentration (Deviche et al., 2012). The time elapsed between sunset on the day of sampling and the time of sample collection was also included as a covariate to account for the relative time of day of sample collection (exact times were not used due to the correlation between season day^2^ and day length). The model included the fixed term ‘status’ (dominant/subordinate) and the random term ‘individual ID’ nested within ‘group ID’ as data in this model included repeated measures from dominant and subordinate males that were from the same group.

A paired within-group analysis was also conducted to examine whether dominance status predicts circulating T, using blood samples collected during the same night from the dominant and a subordinate male in each of 14 social groups (so as to control for variance in T levels arising from either the day or location of sampling).

A LMM was used to investigate whether subordinate male age predicts circulating T concentration. In most cases, male age (years) was assigned from known values of from the date of hatching to the date of sampling. In a sub-sample of cases, the hatching date was not known but the date of fledging was, therefore age was estimated by adding 30 days (the maximum duration for an individual to fledge) to the difference between fledging date and sampling date. Again, T values were square-root transformed for analysis and the significant effect of season day^2^ was controlled for.

#### 2.6.3 Does circulating T predict song characteristics in dominant males?

Three LMMs were used to investigate whether a dominant male’s circulating T concentration predicts three performance characteristics of his solo song (performance duration, syllable rate and proportion of time spent singing). Syllable rate and proportion of time spent singing were square-root transformed prior to analysis. All measures of circulating T and dawn song performance characteristics were collected on the same day for a given male (dawn song in the morning, with capture for blood sampling on the ensuing night; see above). The models included the random term ‘individual ID’ to account for repeated measures of the same male.

The above analyses were conducted to establish whether among-individual variation in T levels predicts among-individual variation in song performance characteristics. We therefore conducted a complementary set of analyses to establish specifically whether natural within-individual variation in circulating T predicts within-individual variation in song performance characteristics, using a sub-sample of nine dominant males for which two sessions of matched dawn song and T measures had been collected within a fortnight of each other in the at the mid-point of the population-level breeding season (the period between season day 100–150). We conducted two within-male analyses for each of the three song parameters (performance duration, syllable rate and proportion of time spent singing). First, we conducted a paired comparison to establish whether the focal song performance characteristic was significantly higher on the day when the male showed their higher T concentration (of the two T measures taken for that male on the different sampling days) than on the day when the male showed their lower T concentration. Second, we conducted a linear regression to investigate whether the within-male change in circulating T from their first to their second sampled day predicted the within-male change in the focal song performance characteristic from the first to the second day.

## 3 Results

### 3.1 Does song production vary in relation to dominance status and timing during season?

**Fig. 3:**
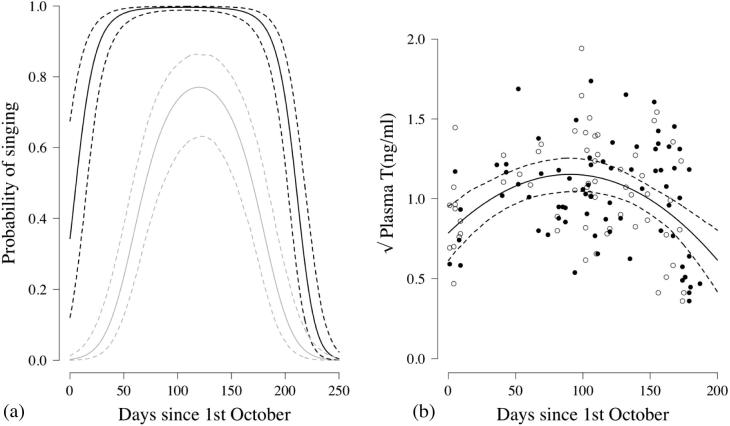
(a) Song production by dominant and subordinate males across the breeding season (commencing 1st October 2010) showing the probability that one or more subordinate males (solid grey line), and the probability that the dominant male (solid black line), in a group sang on a given morning, dashed lines indicate 95% CIs (using GLMM predictions, based on data from 216 dawn observation sessions at 24 groups containing at least one subordinate male); (b) plasma testosterone concentration (square root-transformed) across the breeding season for subordinate (open circles, n = 61 samples from n = 37 males) and dominant males (black circles, n = 72 samples from n = 40 males; solid black line represents predictions from LMM, dashed lines represent 95% CIs).

The probability of song production was significantly predicted by an interaction between dominance status and season day^2^ (GLMM: χ^2^_1_ = 50.83, n = 432 observations from 216 sessions at 24 groups, p < 0.001; Fig. 3a). The probability of song production peaked for both classes halfway through the population-level breeding season, but dominant males were differentially more likely than subordinates to sing towards the start and end of the breeding season, indicating that the magnitude of the effect of dominance status on the probability of song production depended on the time in the season (Fig. 3a). This result held when data collected during within-group egg laying were excluded (χ^2^_1_ = 11.46, n = 400 observations from 200 sessions at 24 groups, p < 0.001).

**Fig. 4:**
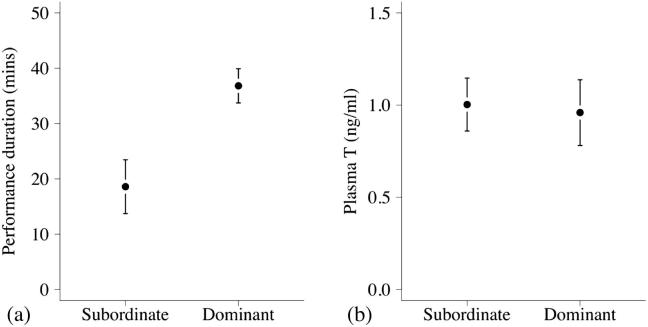
(a) Dawn song performance duration for subordinate (n = 10) and dominant (n = 10) males; and (b) circulating testosterone concentration (ng/ml) of subordinate (n = 14) and dominant (n = 14) males. Males were sampled as matched pairs (1 dominant and 1 subordinate male) from the same group, on the same day. Shown are mean ± SE.

In addition, a matched-pairs comparison of dominant and subordinate male song performances sampled on the same day and in the same social group and outside of the within-group laying period showed that dominant males sang for significantly longer than subordinates on days when both males sang (paired *t*-test: t = 4.79, n = 10 paired males, p < 0.001; Fig. 4a).

### 3.2 Does circulating T vary in relation to dominance status and timing during season?

There was a significant quadratic relationship between season day and the circulating T concentrations of males (LMM: χ^2^_1_ = 22.30, n = 133 samples from 77 individuals, p < 0.001; Fig. 3b); with circulating T concentration peaking halfway through the population-level breeding season. Across the population, there was no significant difference in the circulating T levels of dominant and subordinate males (χ^2^_1_ = 0.43, n = 133 samples (40 dominants and 38 subordinates), p = 0.51; Fig. 3b). None of the other terms included in the maximal model were statistically significant (dominance status × season day^2^: χ^2^_1_ = 0.11, p = 0.74; dominance status × season day: χ^2^_1_ = 1.08, p = 0.29; capture-collection lag: χ^2^_1_ = 0.12, p = 0.73; time of sample collection: χ^2^_1_ = 1.03, p = 0.31). The lack of significant difference in the circulating T levels of dominant and subordinate males still held when data collected during egg laying were excluded from the original dataset and the same model rerun with the reduced dataset (χ^2^_1_ = 0.81, n = 107 samples from 64 individuals, p = 0.36), while the significant quadratic relationship between season day and the circulating T concentrations also remained (χ^2^_1_ = 21.17, p < 0.001). In addition, there was no significant difference between circulating T in relation to dominance status for the samples collected during egg laying (two sample *t* test: t = 0.216, n = 26 samples (13 from 13 dominant individuals (mean = 1.18 ng/ml) and 13 from 12 subordinate individuals (mean = 1.13 ng/ml)), p = 0.83). Furthermore, a matched-pair comparison of dominant and subordinate males from the same social group sampled on the same day, outside of the within-group laying period, revealed no significant dominance-related difference in circulating T levels (paired *t*-test: t = −0.45, n = 14 pairs of males, p = 0.66, Fig. 4b).

There was also no significant effect of subordinate male age (LMM: χ^2^_1_ = 0.0037, n = 61 samples from 38 individuals, p = 0.95) on circulating T concentration, while controlling for the significant quadratic effect of season day (χ^2^_1_ = 12.18; p < 0.001).

### 3.3 Does circulating T predict the song performance characteristics of dominant males?

**Fig. 5:**
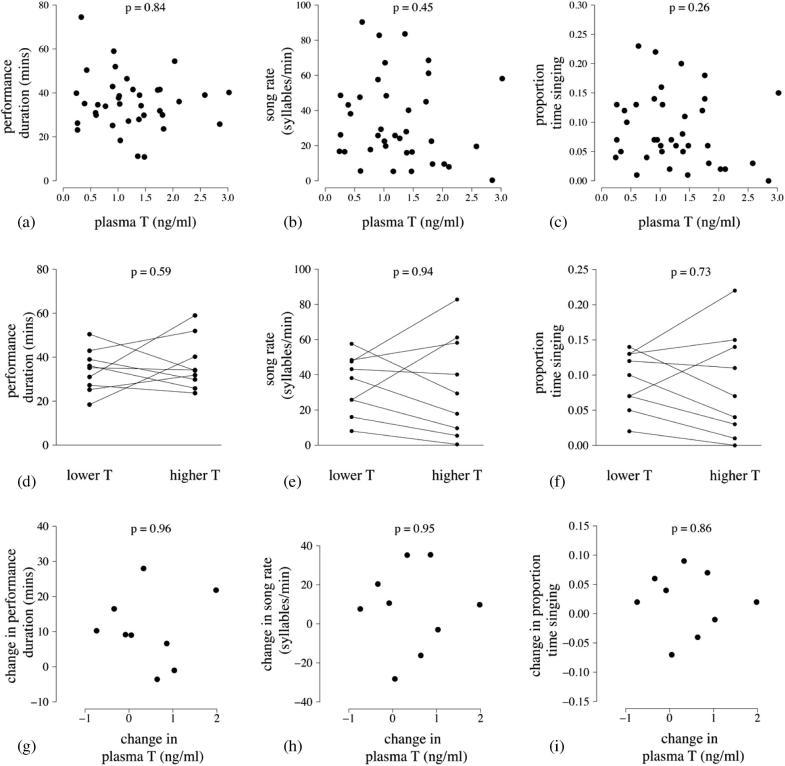
Across-male correlation (n = 28 dominant males; 37 matched song and plasma samples) of T and (a) song performance duration, (b) song rate (syllables/min), and (c) proportion of time spent singing; within-male difference (n = 9 dominant males) from the lower to the higher circulating T session in (d) dawn song performance duration; (e) song rate (syllables/min); and (f) proportion of time spent singing; within-male change (n = 9 dominant males) in T between the two matched sample sessions and within-male change in (g) dawn song performance duration (min); (h) song rate (syllables/min); and (i) proportion of time spent singing.

Across all sampled dominant males (n = 37 matched T and song measures from the same day from n = 28 dominant males), dawn song performance duration was not significantly predicted by their matched circulating T concentration (LMM: χ^2^_1_ = 0.04, p = 0.84; Fig. 5a) when controlling for the significant effect of season day (χ^2^_1_ = 11.10, p < 0.001). Interaction terms between season day^2^ and T (χ^2^_1_ = 0.06, p = 0.80) and season day and T (χ^2^_1_ = 0.01, p = 0.91) were not significant, and neither was season day^2^ (χ^2^_1_ = 2.30, p = 0.13), as would be expected given that the matched sampling of song and T was carried out only during the peak breeding months.

Circulating T concentrations were also not a significant predictor of syllable rate (LMM: χ^2^_1_ = 0.58, p = 0.45, Fig. 5b) or the proportion of time spent singing (χ^2^_1_ = 1.27, p = 0.26, Fig. 5c). None of the other terms included in either model were statistically significant for either syllable rate (T × season day^2^: χ^2^_1_ = 0.90, p = 0.34; T × season day: χ^2^_1_ = 0.85, p = 0.36; season day^2^: χ^2^_1_ = 0.08 p = 0.77; season day: χ^2^_1_ = 0.16, p = 0.69) or proportion of time singing (T × season day^2^: χ^2^_1_ = 1.06, p = 0.30; T × season day: χ^2^_1_ = 0.85, p = 0.36; season day^2^: χ^2^_1_ = 0.12, p = 0.73; season day: χ^2^_1_ = 0.32, p = 0.57).

Comparisons of the song performance characteristics of dominant males repeat sampled in lower- and higher-T contexts revealed no significant within-male differences between the two contexts in any of the focal dawn song characteristics: dawn song performance duration (paired *t*-test: t = −0.56, n = 9 males, p = 0.59, Fig. 5d); syllable rate (t = 0.081, n = 9 males, p = 0.94, Fig. 5e); proportion of time spent singing (t = 0.36, n = 9 males, p = 0.73, Fig. 5f). Nor did the magnitude of the within-male change in circulating T between the two sampling sessions significantly predict the concomitant within-male change in dawn song characteristics (linear regression of change in circulating T against: change in performance duration: t = 0.050, n = 9 males, p = 0.96, Fig. 5g; change in syllable rate: t = 0.066, n = 9 males, p = 0.95, Fig. 5h; change in the proportion of time spent singing: t = −0.177, n = 9 males, p = 0.86, Fig. 5i).

## 4 Discussion

In this study, we investigated whether among- and within-male variation in song production and performance characteristics in a subtropical population of white-browed sparrow weavers was predicted by their circulating levels of T, as would be predicted if natural variation in song production is achieved principally via variation in circulating T. Male song production and circulating T varied in a comparable way with timing in the breeding season, as has been reported for northern temperate species (Foerster et al., 2002, Van Duyse et al., 2003), but in contrast to the seasonal decoupling of male dawn song and circulating T observed in an equatorial songbird (Quispe et al., 2016). However, dominant males were more likely to sing than subordinate males in all months of sampling and sang longer performances than their subordinates in paired within-group comparisons that rule out social environment and territory confounds, despite having comparable levels of circulating T to subordinates in both population-wide and again, in paired within-group comparisons. We also found no evidence that natural variation in the focal song performance characteristics of dominant males (performance duration, syllable rate and proportion of time spent singing) were predicted by their levels of circulating T in either among- or within-individual comparisons. Together these findings suggest that the natural variation in male song performance observed in this population is not achieved principally via variation in circulating T concentration.

Our results contrast with those from a previous study of a more northerly population of this subspecies in Zimbabwe, in which subordinates were never documented to sing and did show significantly lower levels of circulating T than dominants (Voigt and Leitner, 2013); though not in all studies (Voigt et al., 2007). All of the findings to-date for this species are consistent with T playing a role in song *acquisition* and *seasonal onset* of song expression. First, circulating T concentrations and song production rise in tandem at the start of the breeding season (Fig. 3, this paper). Second, experimentally-induced T elevations are sufficient to induce the production of male solo song by females (Voigt and Leitner, 2013). Third, subordinate males have lower circulating T levels than dominants in the population in which subordinates never produce song (the Zimbabwean population; Voigt et al., 2007) but comparable T levels to dominants in the population in which subordinates do produce song (our South African population; this study). However, our findings that (i) dominants exceeded subordinates in both the probability and duration of song despite having comparable levels of circulating T, and (ii) that natural variation in the circulating T levels of dominant males did not predict variation in any of the focal song metrics in either among- or within-male comparisons, are not consistent with the hypothesis that variation in circulating T concentration is the key regulator of *within- and across-male variation* in song performance in this species.

It is conceivable, despite the weight of evidence from both among- and within-individual comparisons in the current study, that circulating T and song output actually are correlated in our study population and that this correlation was not detected because we could not catch males to sample circulating T actually while they were producing song. However, failure to detect such a correlation cannot be readily attributed to circadian variation in mean T levels between the time of song production and blood sampling, as we found no evidence of a systematic change in circulating T levels with sample timing while males were roosting overnight, and the circulating T levels of males sampled in the late evening were comparable to those of males sampled on the following dawn (prior to dawn song production). Consistent circadian variation in T was also not found in previous studies of this species (Wingfield et al., 1991, Voigt et al., 2007). Moreover, previous studies of other species have managed to identify positive correlations between circulating T and song production characteristics, despite time lags ranging from 24 h up to 14 days between song sampling and blood sampling (Foerster et al., 2002, Galeotti et al., 1997, Johnsen, 1998). Consequently, while it is difficult to rule out comprehensively a role for circulating T in regulating song output, our findings certainly suggest a need to consider additional and/or alternative mechanisms that may account for variation in song production.

It is possible that the observed variation in male song production and performance characteristics in our population may be attributable to variation in other components of T-mediated endocrine pathways such as hormone receptor densities. There could be among-individual variation in the densities of T (and/or E2) receptors in relevant brain areas, leading to individual variation in sensitivity to circulating T and thus the effectiveness of circulating T in enhancing song production. For example, if dominant males had more numerous T receptors in the brain centres (HVC, RA) associated with song production this might account for their higher levels of song production despite comparable levels of circulating T. It is therefore notable that a previous study of the Zimbabwean population of this subspecies found that *subordinate* males actually had higher mRNA expression levels for both androgen receptor (AR) and oestrogen receptor (ER) in their HVCs, than dominant males (despite no difference in HVC cell density between the classes), running counter to the rank-related pattern that might otherwise have been expected (Voigt et al., 2007). Although, it is important to note that mRNA levels are not always strong predictors of final protein levels and receptor expression density (Gygi et al., 1998, Quispe et al., 2016). Our within-male comparisons also failed to detect correlations between within-male changes in circulating T and within-male changes in song production characteristics over time, suggesting that reconciling our findings with a key role for circulating T would require one not only to invoke among-male differences in key components of endocrine pathways (such as T receptor levels), but also within-male changes in such components over time. Indeed, in equatorial silver-beaked tanagers (*Ramphocelus carbo*) seasonality in song expression appears to be associated with seasonality in androgen receptor expression in HVC, although within-individual changes in AR expression remain to be demonstrated (Quispe et al., 2016).

Perhaps a more likely explanation for the absence of any relationship between circulating T and dominance status-related song production is that more complex mechanisms than a simple positive effect of circulating T underlie the regulation of male song behaviour in this species (Soma et al., 2008, London et al., 2009). Indeed, castration studies have demonstrated that a number of aspects of singing behaviour are not under control of circulating testosterone alone, for example in male European starlings non-courtship song does not appear to be regulated by testosterone while courtship song is (Pinxten et al., 2002). At least two key additional mechanisms could be at play. First, circulating T (and other circulating androgens) can be locally converted to oestrogen, by aromatase, which may then regulate the expression of song behaviour by binding to oestrogen receptors in the brain (Soma et al., 2008, Remage-Healey et al., 2009). Such an indirect mechanism of action by circulating T could explain our findings, if marked variation in aromatase expression and/or oestrogen reception existed both among and within males. Indeed, such a mechanism would also be consistent with the findings of the many experiments in which manipulating circulating T impacts song production in temperate birds (Table 1), because increasing circulating T would also be expected to alter local oestrogen concentrations within target cells if aromatase expression levels remained unchanged. Second, testosterone and other steroids are also locally synthesised in the brain, via neurosteroidogenesis, and so the actual concentration of a given steroid to which receptors are exposed may reflect a combination of steroids that originate both centrally and peripherally (Soma et al., 2008, London et al., 2009, Remage-Healey et al., 2010). Together, a key role for these neuroendocrine mechanisms in the regulation of song production could readily account for the lack of an evident relationship between circulating T concentrations and song production in this and other studies.

As comparatively few studies (even of temperate species) have considered the role that these alternative neuroendocrine mechanisms may play in the regulation of song production (Soma et al., 1999, Cordes et al., 2014), it is still too early to assess whether the endocrine or neuroendocrine basis for regulating the production of male song is conserved or divergent across songbird species worldwide. Nevertheless, it is notable that the experimental elevation of circulating T in non-temperate superb fairy wrens (*Malurus cyaneus*) did not result in greater song production relative to controls (Peters, 2002), a pattern that contrasts with the typical outcome of this manipulation in temperate birds (Table 1). While both superb fairy wrens and white-browed sparrow weavers are cooperative breeders, it is more likely that the lack of response to circulating T manipulation in superb fairy wrens is due to latitudinal variation in the role of circulating T in relation to song production, given that white-browed sparrow weavers are cooperative breeders throughout their range, but circulating T varies with latitude in this species (see Fig. 1), providing a a potential system for more detailed latitudinal contrasts in future. However, on closer inspection, it is also notable that the evidence for relationships between natural song production and circulating T even in temperate species is mixed (Table 1), highlighting the possibility that the alternative neuroendocrine mechanisms outlined above could conceivably play a wider role in the regulation of song production even in temperate species than is currently widely appreciated. Future studies of song regulation in wild birds might therefore fruitfully combine both correlative analysis of the relationship between natural circulating T and song production with refined experimental manipulations, in an attempt to tease apart the relative contributions of central and peripheral sources of androgens and oestrogens to the regulation of male song production.

## Figures and Tables

**Table 1:** Studies that examine the role of circulating testosterone in relation to song production in free-living male birds during breeding periods. All species breed in northern-temperate habitats except for those indicated: ^∗^subtropical/tropical breeders.

Type of investigation	Species	Song characteristics analysed with respect to T	Song characteristic and T correlated?	Experimental support for role of T?	References
Correlative studies relating natural song characteristics to natural circulating T	Barn swallow (*Hirundo rustica*)	Song rate	No	No Experiment	Saino and Moller (1995)
Song rate Syllable repertoire size Rattle duration Number of impulses per rattle	No No Yes (positive relationship) Yes (positive relationship)	Galeotti et al. (1997)
Blue tit (*Cyanistes caeruleus*)	Song output during dawn chorus Strophe length Song versatility	Yes (positive relationship) No No	Foerster et al. (2002)
European starling (*Sturnus vulgaris*)	Song activity	No	Pinxten et al. (2007)
Red-winged blackbird (*Agelaius phoeniceus*)	Number of songs Number of songs with epaulet exposed	No Yes (positive relationship)	Johnsen (1998)
Song sparrow (*Melospiza melodia*)	% of soft song in non-breeding % of soft song in breeding (song data collected following response to simulated territorial intrusions, not baseline song)	Yes (negative relationship) No	Maddison et al. (2012)
Experimental studies that *elevated* circulating T Experimental studies that elevated circulating T	Blue tit (*Cyanistes caeruleus*)	Song activity	No correlative analysis of pre-manipulation song characteristics and T	Yes	Kurvers et al. (2008)
Diurnal song activity *Dawn song:* Onset Total duration Mean strophe length Mean pause length Mean % performance time Rate Versatility	No No No No No No No No	Kunc et al. (2006)
Dark-eyed junco (*Junco hyemalis*)	Song rate	Yes	Ketterson et al. (1992)
European starling (*Sturnus vulgaris*)	% time spent singing	Yes	De Ridder et al. (2000)
Great tit (*Parus major*)	Song activity	No correlative analysis of pre-manipulation song characteristics and T	Yes	Van Duyse et al. (2000)
Spontaneous song activity Song activity in response to decoy	Yes No	Van Duyse et al. (2002)
Chestnut-collared longspur (*Calcarius ornatu)*	Aerial song display rate	Yes	Lynn et al. (2002)
Lapland longspur (*Calcarius lapponicus*)	Spontaneous song activity Song activity in response to decoy	Yes No	Hunt et al. (1997)
Pied flycatcher (*Ficedula hypoleuca*)	Song rate	Yes	Silverin (1980)
Superb fairy-wren^∗^ (*Malurus cyaneus*)	Song activity	No	Peters (2002)
Experimental studies that *inhibited* the actions of T	Great tit (*Parus major*)	Dawn song: Total duration singing Strophe length Song rate Repertoire size Drift	No correlative analysis of pre-manipulation song characteristics and T	Yes No No No No	Van Duyse et al. (2005)
Blue-headed vireo (*Vireo solitarius*)	Time spent singing Song rate	Yes Yes	Van Roo (2004)
